# Nurses’ challenges for disaster response: a qualitative study

**DOI:** 10.1186/s12873-023-00921-8

**Published:** 2024-01-03

**Authors:** Jamileh Farokhzadian, Parvin Mangolian Shahrbabaki, Hojjat Farahmandnia, Gülcan Taskiran Eskici, Faezeh Soltani Goki

**Affiliations:** 1 Nursing Research Center, Kerman University of Medical Sciences, Kerman, Iran; 2 Health in Disasters and Emergencies Research Center, Institute for Futures Studies in Health, Kerman University of Medical Sciences, Kerman, Iran; 3 Department of Nursing Administration, Faculty of Health Sciences, Ondokuz Mayis University, Samsun, Turkey

**Keywords:** Disaster nursing, Nurses, Response phase, Qualitative research

## Abstract

**Background:**

Healthcare providers, particularly nurses, play a critical role in mitigating the impact of disasters on victims and the healthcare system. However, nurses face unique challenges in disasters that may not experience in their daily practice, which can make it harder for them to deal with disasters efficiently. This study aimed to investigate the challenges faced by nurses for disaster response.

**Methods:**

A qualitative content analysis approach was used in this study. Purposeful sampling was used to select 24 nurses working in the emergency departments of hospitals in Kerman, southeastern Iran. Data were collected through semi-structured interviews and analyzed using MAXQDA10. The conventional content analysis method proposed by Graneheim and Lundman was used to analyze the data.

**Results:**

The analysis of the findings resulted in one major category, insufficient ability of nurses to respond to disasters, and five subcategories: diverse nursing conditions during disasters, inappropriate interactive platform during disasters, the presence of obstacles to teamwork, lack of platform for nurses to acquire adequate disaster risk management competence, and moral tension in complex disaster situations.

**Conclusions:**

Determining the challenges that nurses face during disasters is essential for improving disaster response efforts, promoting disaster preparedness, ensuring appropriate care for patients, and reducing emotional fatigue among nurses. Finally, nursing leaders, healthcare policymakers and governments should use these findings to better support the nursing workforce in disasters.

## Introduction

Incidents and disasters are sudden, unpredictable, and unexpected occurrences that often happen without warning [1]. In recent years, the frequency and severity of disasters have significantly increased. During disasters, there is an imbalance between the needs and the available resources, leading to physical, material, economic, and environmental damages that surpass the society’s existing capacities [2]. Although all the impacts and damages of a disaster are significant, the death, disabilities, and injuries are the most critical [3]. The primary demand of people in disasters is to receive timely and effective health services through proper disaster risk management [4].

Disaster risk management is an ongoing process within the healthcare system that aims to maximize organizational, operational, and skill capacities [5]. Disaster risk management is a process of organizing and directing resources to cope with a disaster, coordinating the roles and responsibilities of responders and to minimize the impact of disaster. This is done by, preventing hazards and minimizing damage, improving preparedness, responding promptly, facilitating recovery [6]. The significant and active involvement of nurses in disaster risk management is undeniable, as they are present during all significant incidents and disasters and encounter various types of incidents [7]. As a crucial member of the healthcare team in all phases of disaster risk management, nurses can play a vital role in reducing the risks, complications, and casualties of disasters through education, management and leadership, counseling, support, guidance, and research [8].

Various studies indicate that, in addition to providing clinical and technical care, nurses require team leadership, problem-solving, creativity, resource management, communication, and teamwork skills in disasters [9–11]. Therefore, nurses need continuous and ongoing development of their skills and competencies for disaster risk management to maintain quality nursing activities before, during, and after disasters [12]. The lack of necessary competencies and preparation of nurses for disaster risk management can increase the adverse consequences of disasters because competent nurses are essential for both the healthcare system and the society [13]. In low-income countries like Iran, these consequences are comparatively higher [14], as the disaster risk management system is not adequately organized and prepared to respond to disasters [15]. Iran is one of the most disaster-prone countries, considering its size, geographical location, and climatic diversity [16].

However, other studies suggest that nurses are inadequately prepared for disaster risk management and lack proficiency in at least one area of disaster risk management [17–19]. During disasters, nurses encounter various challenges such as psychological distress, job stress, health issues, family concerns, organizational issues, poor coordination, and low public awareness [20]. Furthermore, they often encounter issues such as lack of prior disaster care experience, poor preparation, inadequate formal training, disaster-specific ethical and legal dilemmas, and unclear roles [21]. These problems drastically alter the working conditions of nurses in hospitals, particularly in disasters [22]. Health managers are also striving to enhance nurses’ skills and preparedness for responding to disasters, with a focus on ensuring safe and effective responses. Therefore, it is crucial to identify factors that can help to reduce the impact of disasters and enable nurses to effectively manage crises [23].

A review of the literature revealed that most studies on disaster risk management by nurses are quantitative, primarily examining the roles, preparation, perception, and knowledge of nurses about their response to disasters or are in the form of interventional and analytical studies [13, 24, 25]. Qualitative studies that explore the experiences of nurses during disasters can provide rich details about the challenges and difficulties that nurses encounter for response to disasters. It is also important to consider the social and cultural context of the healthcare system in which they work [14]. As the number of disasters continues to rise, and given the limited research on the challenges that nurses encounter in disaster risk management, it is essential to conduct further studies to address this gap. The aim of this study was to explore the challenges that nurses faced for disaster response.

## Methods

### Study design

Conventional qualitative content analysis was used in this study. Conventional qualitative content analysis is an inductive method of qualitative research that involves developing codes, categories and themes from textual data like news articles, research papers, poems, and transcribed interviews [26]. Qualitative content analysis is based on a philosophical framework that emphasizes the importance of context, meaning, and interpretation in understanding human behavior and social phenomena [27]. Conventional content analysis is useful when researchers want to describe a new phenomenon they are investigating or when researchers want to understand the unique lived experiences of a social setting without imposing preconceived theories or categories [28]. Content analysis is particularly useful when the research question involves understanding the meaning, themes, or patterns in the data, as it provides a structured and replicable way to identify and categorize these elements [29].

### Participants and setting

The study was conducted in the emergency departments(ED) of hospitals affiliated with Kerman University of Medical Sciences in southeastern Iran, where care is provided during disasters. Therefore, the first data-rich participants were selected from ED. The selection of the next interviewees proceeded according to the analysis of the interviews. The study used a purposeful sampling method, and a total of 24 participants (13 women, 11 men) were included in the study from March 2022 to April 2023 (Table 1). The study participants were nurses who held varying job positions and had prior experience providing care during disasters. They were selected based on their willingness to share their experiences and insights with the researchers. To ensure the maximum diversity, nurses with different educational levels, work experiences, genders, marital statuses, ages, and job positions were selected to participate in the study.

**Table 1:** Examples of question

1-Can you describe a shift you worked during a disaster?
2-What are the barriers to responding to the needs of those who need assistance in disaster situations?
3-What do you see as the main challenge for nurses to effectively respond to disasters?
4-Do you think the role of nurses may change during a disaster response compared to their usual roles?
5-What kind of training or preparation do you think nurses need to be ready to deal with disasters?
6-What recommendations do you have to improve nurses’ ability to respond to disasters in the future?
7-How do you define an effective disaster response from a nurse’s perspective? What are the key components?

### Data collection

Data were collected through semi-structured in-depth face-to-face interviews. Nurse managers of the hospitals were coordinated and consulted to select eligible participants, and the date and location for the interviews were determined through mutual agreement. A list of general questions was prepared by the researcher in line with the study’s objectives, and additional questions were added during the interview based on the obtained data. To start the study, the researcher schemed an open and general question to encourage the participants to express the first experiences they have in their mind. It also provided a framework for formulating next questions. Table 1 shows examples of the questions that reflected the participants’ experiences of their challenges to respond to disasters. Follow-up questions such as “Can you explain more?” or “Can you give an example?” and “What do you mean?” were asked to obtain more in-depth information based on the participants’ responses. Data collection continued until saturation was reached. All interviews were conducted by the first author, who had sufficient experience in conducting interviews. The interviews lasted between 45 and 90 min, and MAXQDA10 was used for coding and extracting categories and themes.

### Data analysis

The approach proposed by Graneheim and Lundman was used for qualitative content analysis [30]. The interviews were digitally recorded, transcribed verbatim, and reviewed, coded, and analyzed. Data analysis was conducted simultaneously and continuously with the data collection process. For initial coding, the researchers used the participants’ statements and indicative codes taken from the interviews. Primary codes were extracted from the interviews as meaningful units from the participants’ statements. The codes were repeatedly reviewed and categorized based on their similarity and proportion. Subsequently, similar codes were merged, and the categories became more complete, forming the second-level coding. In the next step, the categories were compared with each other, and those with similar characteristics were combined to create broader categories. The codes obtained from the data analysis were continuously reviewed and corrected until the final stages of research writing. The primary extracted codes were reduced during continuous data analysis and comparison, and the main theme, sub-categories, and categories regarding nurses’ experiences for response to disasters were finalized. All study authors had experience working in disasters. This work experience helped as a road map to develop and guide the study and identify the gaps and shortcomings. The researchers were aware of the potential impact of their experiences on the study. So, by using reflective notebook, consulting with experienced researchers and focusing on the voice of the participants, the researchers tried to control the influence of their own reflection or experience in the research and ensure that their personal experiences and biases did not unduly influence the results of the study.

### Trustworthiness

To ensure the data trustworthiness, the Guba and Lincoln criteria (credibility, dependability, confirmability, and transferability) were used [31]. Moreover, member check was used in addition to prolonged involvement of the researcher to increase the credibility of the data. Also, after encoding, the interview transcripts were returned to the participants to ensure the accuracy of the codes and the relevant interpretations. Peer check approach was used to control for data confirmability; for this purpose, the data were coded and categorized which were later evaluated by the research team. When there was no consensus between codes and categories, discussion was continued to clarify the issue and to reach a consensus. To control for the dependability of the data, an audit trail was used. In the implementation of this method, the researcher maintains the preliminary data, categories, and themes until the end of the research process. Moreover, the review and analysis of the data of the experienced individuals in the research by two qualitative researchers outside the study increased the reliability of the study. The transferability of the study also depended on the evaluation and approval of findings of the individuals in the same environment. Sampling with a maximum variance also helped in the transferability and stability of the results, as well as credibility of the data. Allocation of sufficient time to the study and face-to-face communication with the participants were other factors that increased the data credibility. Results were also confirmed by some non-participant nurses.

### Ethical considerations

This research was approved by the Ethics Committee of Kerman University of Medical Sciences (IR.KMU.REC.1401.188), and permission to conduct the study was obtained. The present study followed the ethical guidelines outlined in the Declaration of Helsinki. The researchers introduced themselves to the participants and clearly explained the study objectives and data collection methods. Written informed consent was obtained for participation in the study and audio recording. The place and time of the interviews were chosen based on the preferences of the participants. The participants were assured of the data confidentiality throughout all stages of the research and were allowed to withdraw from the study at any time. Additionally, participants were encouraged to contact the researchers with any questions or concerns they may have had.

## Findings

Table 2 shows the demographic information of the participants in this study. Of the 24 nurses participating in the study, 11 were male nurses, 18 were married, and 17 had a bachelor’s degree. The job position of the participants included 16 nurses, 1 nursing manager, 3 supervisors, and 4 head nurse. Their age range was 34–55 years and their average work experience was 17.5 years.

**Table 2:** Characteristics ‘participants (*N* = 24)

Participant’s code	Work experience (Years)	Level of Education
1	20	Bachelor’s degree
2	25	Bachelor’s degree
3	10	Bachelor’s degree
4	26	Bachelor’s degree
5	10	Master’s degree
6	10	Bachelor’s degree
7	22	Bachelor’s degree
8	23	Bachelor’s degree
9	25	Bachelor’s degree
10	20	Bachelor’s degree
11	10	Bachelor’s degree
12	18	Bachelor’s degree
13	22	Master’s degree
14	8	Master’s degree
15	20	Bachelor’s degree
16	15	Bachelor’s degree
17	10	Bachelor’s degree
18	17	Master’s degree
19	19	Bachelor’s degree
20	12	Bachelor’s degree
21	20	Master’s degree
22	19	Master’s degree
23	18	Bachelor’s degree
24	23	Master’s degree

Through the analysis of the interviews, the researchers identified a main theme: insufficient ability of nurses to respond to disasters. The analysis also revealed five categories: diverse nursing conditions during disasters, inappropriate interactive platform during disasters, the presence of obstacles to teamwork, lack of platform for nurses to acquire disaster response competencies, and moral tension in complex disaster situations (Table 3). Each category consisted of subcategories, which were explained below with quotations from the participants.

**Table 3:** Themes, categories and subcategories extracted from qualitative content analysis

Theme	Categories	Subcategories
**Insufficient ability of nurses to respond to disasters**	Diverse nursing conditions during disasters	• Confusion and uncertainty during disasters • The unknown end of disasters • Intensifying cognitive and psychological impacts of job during disasters
	Inappropriate interactive platform during disasters	• Challenges of communicating the reality of disaster conditions to companions • Controlling emotions inappropriately as a factor in unfavorable interactions • Communication without support
	The presence of obstacles to teamwork	• Lack of commitment to collaborative management among nurse managers • Development and monthly implementation of schedules in nursing practice • Management of inexperience among novice nurses during disasters • Lack of disaster risk management experience among nurse managers
	Lack of platform for nurses to acquire adequate disaster risk management competence	• Lack of continuous disaster risk management training for nurses • Lack of effective disaster risk management training among nurses • Lack of equipment for practical training • Difficulty in determining and evaluating disaster risk management competencies • Lack of development and implementation of evidence-based clinical guidelines
	Moral tension in complex disaster situations	• Dilemma between personal health and sacrifice in nursing practice during disasters • Dilemma between family responsibilities and voluntary work in nursing practice • Making effective decision in resource-limited situations • Inadequate disaster preparedness in nursing practice • Complexity of effective triage protocols in nursing practice during disasters

### Diverse nursing conditions during disasters

During disasters, nurses experience diverse and different roles compared to routine situations. They face an increase in the number of patients and require a much broader level of care and resources. Nurses experience prolonged presence in the work environment, sudden changes of work routines, and find themselves in an unfamiliar environment. Additionally, nurses do not know when the situation will return to normal, and providing care requires a quick response, which increases their vulnerability in the work environment. These are permanent characteristics of disasters, and nurses experience disasters to varying degrees each time they are exposed to them. Nurses described their experiences as confusion and uncertainty during disasters, the unknown end of disasters and intensifying cognitive and psychological impacts of job during disasters.

### Confusion and uncertainty during disasters

According to the participants, the lack of information and uncertainty regarding how to treat and care for patients, unexpected situations, the confusion of the work environment, the chaos and crowding, and the presence of contradictory information about how to provide care during disasters, resulted in feelings of confusion and worry. Nurses described these conditions as chaotic scenes during disasters, which made it difficult for them to make correct decisions and provide quality care. So, they may provide care and treatments with doubt and uncertainty regarding their effectiveness.

*“I was unsure of what was right or wrong when admitting patients with alcohol poisoning, despite having received numerous training courses. The situation was chaotic and disorganized. In addition to my responsibilities as an operator and receptionist, I had to act as a secretary and liaison between several doctors.” (P3)*.

*“On that day, even the doctor was unsure which approach was the best for the patients who were airlifted in. Additionally, my colleague was inexperienced in triage and was learning in that situation.” (P15)*.

### The unknown end of disasters

The nature of disasters makes it difficult to predict their duration or how they will end. Nurses expressed negative impacts of the uncertain end and the long duration of disasters. It is unclear how to accelerate and improve care. The unknown duration and process of disasters caused anxiety and fatigue, and led some nurses to leave their job.

*“Two of my colleagues and I went to provide care to the victims of the Kermanshah earthquake. During that time, I constantly asked myself the question: when will this finally end? It was the unanswered question that bothered me.” (P10).*

*“The nurse came voluntarily. During the first few days, she worked energetically, hoping that it would end soon. However, after a while, she could not continue. She became frustrated and made excuses until she finally gave up and left.” (P1)*.

### Intensifying cognitive and psychological impacts of job during disasters

Nurses’ physical and mental vulnerability in the workplace is amplified during disasters. They may experience irritability, difficulty sleeping, intrusive thoughts, reduced activity levels, emotional numbness, physiological reactions, memory impairment, and post-traumatic stress disorder (PTSD). Additionally, they often care for people infected with contagious diseases, leading to fears of infecting themselves and their families. These injuries are exacerbated by the constant exposure to injured people and victims of disasters, and nurses may experience job burnout, job loss, inefficiency, and physical and mental illness. It is crucial for them to receive adequate support to cope with these challenges.

*“While providing care to the victims of the Bam earthquake, I was constantly on the go and never had a break. My body was in pain, and even when I tried to rest, the haunting scenes of the victims kept me awake. Even today, I still cannot bring myself to return to Bam. I have been experiencing symptoms of depression since then, and it continues to affect me.” (P8)*.

*“After four days of working on disaster relief, I returned home feeling drained and unable to connect with others. My son repeatedly urged me to quit my job. Although I was initially hesitant, I eventually decided to take a break from work.” (P14)*.

### Inappropriate interactive platform during disasters

During disasters, nurses must communicate with healthcare team members, clients, and their families. As a result, effective communication becomes more complex and challenging. Nurses may struggle with choosing the right words and phrases to provide information, empathizing with clients, and gaining their trust in disaster conditions. Additionally, the lack of time and emotional imbalances can lead to inappropriate interactions with the healthcare team, clients, and their companions. Managing interactions becomes a difficult and stressful task. Experienced nurses can rely on their extensive knowledge to manage interactions and serve as supporters for other nurses. This category included subcategories such as challenges of communicating the reality of disaster conditions to companions, controlling emotions inappropriately as a factor in unfavorable interactions, and communication without support.

### Challenges of communicating the reality of disaster conditions to companions

Nurses were concerned about their interactions with the companions of injured patients during disasters. Confusion, emotional instability, misplaced expectations, and lack of information about the current conditions caused companions to have an inaccurate understanding of the situation. These issues can disrupt the provision of care, waste time, and increase nurses’ stress levels. As a result, the communication between nurses, patients, and their companions became distorted, making it difficult for nurses to manage interactions. While nurses tried to establish more effective relationships with families and companions through training and providing sufficient information, they might not always be successful.

*“On that day, emotions were running high among the patients’ companions. Some were crying, others were angry, and some were confused. They all expected me to prioritize their loved one’s care above others. Unfortunately, only a few were obedient and cooperative. However, the inappropriate presence of the companions made it challenging to provide care and consumed a significant amount of my time and energy. These situations led to verbal and physical conflicts between me and the companions.” (P23)*.

*“The parents of the child were confused and did not know how to react in the tense situation. The flood had destroyed their entire life, and on top of that, their child was experiencing shortness of breath. I tried to calm them down by speaking to them, but it was challenging to focus on my work.” (P12)*.

### Controlling emotions inappropriately as a factor in unfavorable interactions

Nurses face emotional and psychological challenges when working in tense situations during disasters. These challenges can manifest as anger, sadness, anxiety, mood disorders, and fear. Nurses who experienced these negative emotions were more likely to have conflicts with their colleagues, managers, and companions. They were also more susceptible to injury, and their desire to leave their jobs was significantly higher during disasters. Improper control of emotions could prevent nurses from communicating effectively with others, leading to adverse interactions, physical and psychological injuries. These findings suggest that nurses may lack the necessary skills to control their emotions in difficult situations such as disasters.

*“Explaining the situation to families and relatives was difficult and stressful for me. I often found myself unsure of what to say and how to say it. There were times when I preferred not to say anything at all because I had not learned how to manage such situations.” (P17)*.

*“The patient’s companion was very angry. He was crying and even physically assaulted me. The experience stayed with me, and I now feel afraid of interacting with companions in similar situations. It has even made me question my decision to become a nurse. I learned how to manage such situations and seek help from others.” (P14)*.

### Communication without support

During disasters, the working conditions for nurses became significantly more challenging. However, at times, managers failed to provide the necessary support, despite having more communication with nurses during emergencies. As a result, the financial, psycho-spiritual, and welfare support for nurses during disaster risk management was often insufficient. This lack of support can lead to nurses losing their motivation and becoming distrustful of their managers, which make it challenging for them to provide care for the injured. By creating a supportive environment, managers can help nurses develop open and honest communication during disasters.

*“I was pregnant and repeatedly requested a transfer from the emergency department in writing and in person to managers. Unfortunately, I did not receive any response, and my manager did not seem to care about solving my problem. This made me unable to have a good relationship with the nursing manager and we argued with each other many times on various issues.” (P5)*.

*“During the COVID-19 pandemic, I met a young and inexperienced nurse who decided to leave the nursing profession due to her experience working in such a critical situation. She confided in me, saying, ‘I am never going back. I always tried to provide the best care for my patients, but I never had the necessary equipment when I needed them. I felt alone, and no manager seemed to understand me.” (P6)*.

### The presence of obstacles to teamwork

Nurses recognized the importance of teamwork and collaboration within their profession. They believed that working together provided an excellent opportunity for them and their managers to ask questions, obtain information, correct misunderstandings, and discuss previous experiences. During disasters, the need for teamwork becomes even more crucial, but with changing circumstances and critical situations, it becomes more challenging for nurses to work effectively as a team. The complex, changing, high-risk, and emergency nature of disasters, along with poor disaster risk management, could hinder cooperation, participation, and teamwork among nurses. This category included subcategories such as lack of commitment to collaborative management among nurse managers, development and monthly implementation of schedules in nursing practice, management of inexperience among novice nurses during disasters, and lack of disaster risk management experience among nurse managers.

### Lack of commitment to collaborative management among nurse managers

Experienced nurses stressed the importance of sharing their experiences from different disasters with nurse managers to better manage teamwork. However, managers were often not consulted regarding decisions and team management and might lack flexibility in leading and managing the teams. It can lead to a reduction in nurses’ motivation to cooperate and participate in teamwork.

*“During my two months of work in the earthquake area, our team provided suggestions to managers on how to improve care for the injured and transfer them to other hospitals. Unfortunately, our recommendations were often ignored, and managers did not consult with us. I recall one instance where my manager told me he did not see the need to consult with the care team.” (P7)*.

### Development and monthly implementation of schedules in nursing practice

Managers, particularly head nurses, faced challenges in adjusting the monthly schedule to meet the demands and capacities of the nursing staff during disasters. The head nurses identified the monthly schedule as a significant challenge for teamwork. However, nurses felt that their head nurses did not understand their needs, while head nurses were forced to complete the schedule with the same number of nurses despite a shortage of available staff and a high workload. This issue weakened the effective interaction between nurses and head nurses.

*“Due to various circumstances such as pregnancy, withdrawal from the plan, sick leave, and requests for leave from experienced nurses, I found myself in constant arguments with the nurses under my supervision while trying to write the monthly schedule. These conditions made the task extremely challenging.” (P13)*.

### Management of inexperience among novice nurses during disasters

Managing new nurses was a significant challenge for nurses at all levels, particularly during disasters. Managers often try to recruit new human resources during or after disasters. However, inexperienced nurses may not be adequately prepared to provide care during such crises, leading to challenges for the care team to perform their tasks effectively.

*“I once worked a shift with a new nurse, and it was a very exhausting experience. The nurse was not able to perform the correct nursing procedures, such as placing a Foley catheter or inserting an NGT tube. I had to constantly assist and guide her, and it felt like I was the only one taking care of the patient.” (P2)*.

*“Working alongside a colleague who was struggling to perform adequately in a critical situation was a source of concern for me. Their poor performance made it challenging to stay focused on my own tasks, and I felt that it posed a risk to both patients and myself. I had to continually monitor their work to ensure that no care was missed and no mistakes were made.” (P9)*.

### Lack of disaster risk management experience among nurse managers

Some nurse managers lacked experience in handling disasters, and even those with past experience were unable to manage new and unique situations effectively. Some nurse managers were not familiar with the plans and processes governing certain situations, potentially related to disaster response or emergency management. As a result, they acquired basic competencies through trial and error. These managers had both positive and negative perceptions of their empowerment in disasters.

*“During the disaster, everything was in disarray, and I found myself praying that no more patients would arrive. Despite my hope, they continued to bring in more injured and sick individuals. Some of the routine procedures had changed, making it challenging for me to provide the necessary care. It took two to three months to get everything under control, overcome the confusion, and manage the situation effectively.” (P9)*.

#### Lack of platform for nurses to acquire adequate disaster risk management competence

The study participants believed that they did not receive the necessary training for disaster risk management in real-life situations. They cited a lack of effective and continuous training as well as insufficient practical training using appropriate equipment. Nurses also needed to participate actively in their learning and maintain motivation for continuous and group training. Additionally, managers should provide support to help nurses acquire the necessary competency in disaster risk management. Developing disaster risk management competencies in nurses requires not only academic training but also the commitment of managers, organizations, and nurses to effective continuous education. The subcategories that emerged from this category included, lack of continuous disaster risk management training for nurses, lack of effective disaster risk management training among nurses, lack of equipment for practical training, difficulty in determining and evaluating disaster risk management competencies, and lack of development and implementation of evidence-based clinical guidelines.

### Lack of continuous disaster risk management training for nurses

Nurses emphasized the importance of their competence and preparation in responding to disasters safely and effectively, particularly in rescuing and caring for the injured. They believed that structured, effective, and continuous training was essential for improving their disaster risk management abilities. Nurses identified the lack of continuous training as the most significant reason for failure to learn skills and acquire disaster risk management competence. These challenges had a significant negative impact on nurses’ practices during disasters.

*“Training courses such as ECG interpretation, cardio-pulmonary resuscitation, and familiarization with how to care for different diseases are repeated frequently throughout the year. However, disaster risk management training is often limited to a one-day workshop or held irregularly and theoretically. Theoretical training could be useful, but it is essential to combine it with practical training in a real or simulated environment to complete our competencies.” (P21)*.

Several nurses reported that their workload and the number of shift work prevented them from attending training sessions regularly.

*“The disaster risk management skills workshop was held in four sessions, but due to my heavy workload, I was only able to attend two of them. Unfortunately, the workshop was not held again, and I missed the remaining sessions. The workshop instructor was from the Tehran Emergency Organization.” (P11)*.

#### Lack of effective disaster risk management training among nurses

Nurses reported the least participation and attendance in disaster risk management training programs. They expressed that their educational needs were often not reflected in disaster risk management training and that the training provided was relatively superficial and not considered a priority in managers’ plans. Neither managers nor nurses prioritized the need for disaster-related training. However, most of the situations created in disasters cannot be experienced in advance. Therefore, nurses may lack motivation to participate in training courses due to the lack of immediate and routine application of the training.

*“As a nurse, I have many other training courses to attend, and attending disaster risk management skills training is not among my highest priorities. Also, managers do not care that we must participate in disaster risk management training throughout the year.” (P10)*.

#### Lack of equipment for practical training

To effectively prepare nurses for disaster risk management, it is crucial to develop their knowledge and skills concurrently. However, in common training courses, only theoretical knowledge of disaster risk management is imparted, and nurses cannot acquire disaster preparedness skills. Practical training related to disaster risk management requires more educational facilities and equipment, and the lack of educational facilities can leave nurses and instructors dissatisfied with educational activities.

*“We did not have complete equipment for our practical training, and the entire session was in the form of lectures. Despite having a good instructor, the lack of facilities made it difficult for us to learn as we should. Many nurses even decided not to participate in these workshops anymore because they believed that practical training was necessary to acquire the skills needed to perform in a real-life situation.” (P18)*.

#### Difficulty in determining and evaluating disaster risk management competencies

The diversity and large number of skills required by nurses for different phases of disaster risk management, as well as the lack of a comprehensive tool to evaluate all competencies, make it difficult and complicated to assess nurses’ readiness for disasters. The knowledge and skills required by nurses can vary based on the type of disaster, resulting in a wide range of necessary skills that are often less evaluated. Additionally, some nurses can only acquire or experience these skills when faced with disaster situations.

*“It is challenging to determine the exact skills I need to learn for disasters. I have attended many disasters, and each time, it felt like a new and unique situation. For instance, taking care of victims of flood, earthquake, chemical accidents, terrorism, and more, each requires different skills and evaluations.” (P21)*.

*“I attend almost all the training courses offered by the continuing education system and the hospital at my workplace. However, it was only during the COVID-19 pandemic, when the incident command system was activated, hospitals were leveled, and other measures were taken, that I realized the need for broader skills and knowledge beyond technical expertise to work during disasters.” (P4)*.

### Lack of development and implementation of evidence-based clinical guidelines

The majority of nurses perceived the importance of using research findings and applying them to their work. However, they have not yet seen sufficient scientific evidence to guide disasters in nursing practice. While they were eager to utilize research results, clinical guidelines, and current technologies, they had not been provided with adequate training in these areas. The nurses expressed a strong desire to increase collaboration and coordination in order to implement evidence-based approaches during disasters.

*“I discovered several practical guidelines in the Ministry of Health Website that I was previously unaware of. These guidelines provided checklists for infection control, CPR processes, triage, and evaluated necessary equipment, physical space, and staffing requirements during disasters. I am grateful to have found these guidelines and will continue to refer to them in my work.” (P12)*.

*“Many nurses lack knowledge of important skills like triage start, jump start, and incident command system, as well as the appropriate dressings for critical situations like disasters. While some advanced countries use mobile phones for injury screening and follow-up during disasters, we have not received any training on this technology or seen any related clinical guidelines.” (P24)*.

### Moral tension in complex disaster situations

Providing care during disasters presents challenging situations, and ethical dilemmas are often unavoidable. For instance, the lack of equipment and medicine could make nurses feel ashamed, guilty, and uncomfortable. Decisions became difficult and sensitive, as nurses struggled to allocate limited resources to benefit all patients equally. They might also struggle to identify which patient should receive treatment first. Insufficient preparation could also lead to feelings of inadequacy and concern that their actions might cause more harm than good, adding to their emotional burden. This category included subcategories such as dilemma between personal health and sacrifice in nursing practice during disasters, dilemma between family responsibilities and voluntary work in nursing practice, making effective decision in resource-limited situations, inadequate disaster preparedness in nursing practice, and complexity of effective triage protocols in nursing practice during disasters.

## Dilemma between personal health and sacrifice in nursing practice during disasters

Nurses faced physical, mental, psychological, and emotional risks when providing care during disasters, leading to moral tensions. Despite these risks, many nurses demonstrated their dedication by providing care in the most difficult and critical disaster situations, even when their own safety was threatened. However, when faced with patients in urgent need of treatment, they might hesitate to continue working due to concerns about their own health. They might find themselves having to choose between their own health and making a sacrifice for the benefit of their patients.

*“As a new nurse, I faced with a difficult decision when my head nurse asked me to provide care for a 24-hour shift during a disaster. The prospect of working such long shifts while facing potential health risks was a cause for concern, and I found myself unsure of what to do in such situations.” (P5)*.

*“During my service in the aftermath of the Kermanshah earthquake, I found myself in a dangerous situation during an aftershock. While I was tense and aware of the potential risks to my own safety, I could not ignore the needs of those around me. I first rescued a child and then went on to lead other patients out of their rooms.” (P13)*.

*“After the alert was announced, we advised nurses with physical health problems or underlying medical conditions not to work in the ward. While some nurses immediately assessed their situation and changed their workplace accordingly, others chose to stay and prepare for the crisis, despite the potential risks to their health. These nurses remained committed to their duties and continued working until the end of the crisis.” (P15)*.

### Dilemma between family responsibilities and voluntary work in nursing practice

Working in critical conditions and being away from their families were painful experiences for nurses. They described this as an unpleasant experience and found it difficult to cope with changes in their family dynamics due to their absence. As a result, family was a major concern for nurses, and it significantly reduced their willingness to work in unconventional conditions such as disasters.

*“Even though I was concerned about my family’s well-being, I knew that I could not be indifferent and simply return home during the disaster. However, I found myself constantly asking questions such as, “Are my family members facing any problems or difficulties?” and “How are they managing their needs in my absence?” These concerns caused me a great deal of anxiety and irritation.” (P19)*.

Married female nurses tended to be more hesitant about taking on professional roles in disasters. They expressed concerns about their personal and family responsibilities, including caring for children and managing household duties, in addition to being pregnant or having an infant. The added challenge of opposition from their husbands further complicated their ability to fulfill their responsibilities.

*“My husband has been insisting that I quit my job, and I find myself contemplating job abandonment during disasters. It’s challenging to balance my work, children, and personal responsibilities and make the best decisions for my family and myself.” (P12)*.

### Making effective decision in resource-limited situations

One of the major concerns of nurses in disasters was the lack of medicine and equipment, which could compromise the quality of care provided to patients. This could be a significant source of stress for nurses, who might feel unable to provide the level of care they would like to. Nurses emphasized the importance of adhering to disaster risk management guidelines and ensuring that care was provided uniformly, despite the scarce resources available. The lack of resources and equipment can have serious consequences, with some high-risk patients not receiving the care they need to survive.

*“There were patients who were not attached to ventilators due to low chances of survival, or who were in pain but lacked access to sedatives. The shortage of nurses in some cases also meant that certain aspects of healthcare could not be provided. These situations were distressing for me, and I wished that there was an abundance of resources to provide better care and avoid having a guilty conscience.” (P10)*.

*“A number of beds were already occupied by patients, and with the influx of injured people, some of the beds were destroyed. We found ourselves in a difficult situation where we had to place two patients in one bed and hope that they could be safely accommodated. This was a difficult moral decision for me to make.” (P13)*.

#### Inadequate Disaster preparedness in nursing practice

Nurses expressed their commitment to individual, organizational, and national preparedness for effective disaster response as a moral obligation. However, at times, nurses also felt incompetent to perform their duties during disasters, which led to feelings of inadequacy and moral concern.

*“Due to lack of work experience in intensive care units during disasters, I felt helpless and morally concerned. Despite theoretical knowledge of how to work with specialized devices, I found myself unable to provide the necessary care to the injured. This created a difficult situation for me, and I felt helpless and guilty.” (P17)*.

*“We experienced delays in receiving operational instructions from our supervisors. For instance, when we received patients with poisoning from Rafsanjan, the supervisor failed to coordinate the transfer of patients or call the on-call team in a timely manner. This delay resulted in significant waste of time, which ultimately had a negative impact on the patients.” (P16)*.

#### Complexity of effective triage protocols in nursing practice during disasters

In disasters, correct and rapid triage is essential for successful operations. Triage errors can worsen critical situations, making them more challenging. Unfortunately, triage nurses often faced uncertainty, fear, doubt, error, and vulnerability in such situations. They had to prioritize patients correctly in a limited time and a stressful environment, with minimal facilities. During disasters, effective triage was considered a fair and necessary practice to allocate limited medical resources to those in urgent need. However, assigning black-label triage status and making difficult decisions based on resource consumption could cause moral distress for nurses who may be forced to prioritize patients based on factors such as the severity of their condition and the likelihood of survival.

*“During disasters, we prioritize patients in urgent need of care, leaving critically ill patients who are expected to die out. This can be morally distressing for nurses as their instinct is to protect and help all patients. Assigning a black label to critically ill patients is particularly difficult.” (P23)*.

Nurses’ ethical decision and prioritization during triage is widely recognized, yet nurse managers may be careless in their approach. In some cases, they fail to use reasonable and scientifically accepted criteria to select triage nurses. Participants in this study reported that the negligence in the selection process was a specific ethical issue that needed to be addressed.

*“During disasters, triage is a crucial task, but nurse managers may not always consider the appropriate qualifications when selecting nurses for this role. In some cases, they may assign triage duties to nurses who are not ideally suited for the task. For instance, during a sudden influx of injured people after a bus collision, a pregnant nurse found herself alone in the triage unit and struggled to manage the situation on her own.” (P15)*.

## Discussion

The aim of this study was to investigate the challenges and problems faced by nurses in disaster response. Nurses often provide care for victims during disasters with minimal support and at great personal risk. Their expectations and responsibilities increase in an unpredictable environment with limited resources, leading to significant challenges in disaster risk management. The main theme identified in this research was the insufficient ability of nurses to respond to disasters, which included subcategories such as diverse nursing conditions during disasters, inappropriate interactive platform during disasters, the presence of obstacles to teamwork, lack of platform for nurses to acquire adequate disaster risk management competence, and moral tension in complex disaster situations.

The present study highlighted inevitable challenges faced by nurses during disasters due to the unpredictable and complex nature of such events. These challenges included fear of the unknown, inexperience in dealing with new situations, making mistakes due to limited and contradictory information, constant stress, and inadequate professional skills. Working conditions during disasters can be high-risk and error-prone, potentially affecting patient safety and care quality [32]. The study findings align with previous research indicating that nurses experienced a stressful work environment during disasters [33, 34]. The study also found that disasters could cause noticeable changes in the work environment, including imbalance, disorganization, and chaos, along with unpredictable conditions, long working hours, limited resources, and unfamiliar environments [20]. These changes complicated the provision of care and led to fatigue, lack of interest in work, and job abandonment [35]. The numerous changes in working conditions and resulting challenges during disasters, particularly in low- and middle-income countries like Iran, can make disaster risk management more challenging for nurses [36].

The present study found that nurses faced inappropriate interactive platforms during disasters, including misunderstandings among companions, difficulty controlling emotions, and a lack of supportive communication. Despite being constantly involved in the physiological, psychosocial, and environmental needs of patients and their families during disasters, managers often increased the workload of nurses without providing significant support. Consequently, nurses worked selflessly and did their best to care for patients, but were treated disrespectfully and even violently by managers, patients, and their companions [37]. Furthermore, the study found that managers provided insufficient support to nurses following injuries and did not understand their personal and family conditions, needs, and demands. These conditions negatively affected the interactions of nurses and made it difficult for them to manage. Xue et al. emphasized that through effective communication, managers could understand the needs of nurses, provide emotional support to them, and help them become more resilient [37]. Similarly, Hou et al. suggested that nurses required professional training to improve their communication with colleagues, managers, and other healthcare members during disasters [38]. Cultural and social factors such as traditions, beliefs, religion, and gender can also influence communication during disasters, and these factors vary across cultures. For instance, in Middle Eastern countries like Iran, clients are highly sensitive to the nurse’s gender and prefer to be cared by nurses of the same gender. Similarly, people with different religions or ethnicities may prefer to receive care from nurses with the same religion or ethnicity, which can pose cultural challenges for nurses during disasters [39].

The present study identified several aspects of managers’ lack of support that negatively affected nurses’ ability to manage disasters, including refusal to listen to their concerns, frequent reprimands in front of others, disregard for their well-being and rest during shift work, indifference to personal demands and problems, and delays in solving work environment deficiencies. According to Xue et al. nurses highlighted the importance of managers’ support in empowering them to respond to disasters effectively [37]. Behaviors such as rewards and verbal appreciation were reported as supportive actions by managers, which is consistent with Iranian culture. Sihvola et al. confirmed that positive feedback could encourage nurses to become more self-confident and autonomous [40]. Working conditions during disasters can be so harmful that nurses may experience psychological and emotional distress and physical injuries. Researchers have reported a close relationship between psychological and physical injuries caused by work and unsuccessful interactions of nurses in the work environment [41]. Nurses in the present study identified psychological injuries and negative emotions as factors that hindered their ability to communicate effectively during disasters. Jiménez-Herrera also suggested that negative feelings might arise from interactions with companions, managers, and other healthcare providers [42]. It is essential to take special measures to prevent the physical and mental-emotional injuries of nurses in the work environment during disasters. This is crucial for improving their interactions during disasters and should be tailored to the structure and context of each society.

Another critical finding of this study was nurses’ teamwork problems during disasters. Factors such as managers’ poor commitment to collaborative management, difficulty in performing monthly schedules, novice nurses’ inexperience, and managers’ insufficient experience in disaster risk management were identified as challenges. During disasters, nurses and managers must work closely together as a team. Effective team leadership and management skills play a critical role in successful disaster risk management [32]. Some participants reported feeling a communication barrier between themselves and their managers, leading to unresolved conflicts. They believed that managers lacked knowledge and experience in leading a team and resolving conflicts and were unwilling to accept suggestions and experiences from other team members. The study found that nurses’ inability to work effectively as a team during disasters could result in mistakes, inappropriate interactions, incompatibility, and fatigue among team members. However, nurses also recognized the benefits of teamwork such as the use of colleagues’ knowledge and experiences from previous disasters, increased job satisfaction, and reduced stress levels [43]. The presence of new nurses during disasters created challenges and concerns for colleagues and managers in successful teamwork. Experienced nurses often have a poor professional image of new nurses and perceive them as a burden. Managers are also under pressure to compensate for the lack of expertise of new nurses. Novice nurses experience fear of making mistakes, inexperience, constant stress, and poor interactions within the care team. These experiences can negatively affect their motivation and hope, and they often perceive a lack of support and empathy from their colleagues and managers. The researchers emphasized the importance of colleagues’ and managers’ support in addressing the challenges faced by novice nurses in teamwork [44].

The study participants had concerns about their ability to respond appropriately to disasters, as they felt that their disaster risk management training was inadequate. The multifaceted and complex nature of disasters also means that there is no valid, comprehensive, and standardized tool to evaluate nurses’ disaster preparedness and qualifications. The researchers emphasize that this issue is not unique to Iran but is a global challenge where disaster education for nurses is often neglected [21]. The type and manner of training are critical in ensuring that nurses achieve disaster risk management competencies [45]. Practical training based on simulation and various maneuvers was identified as the most effective way to teach disaster risk management skills to nurses [20, 46]. However, some studies showed deficiencies in the training provided for disaster risk management preparation, resulting in nurses’ dissatisfaction with the training and their insufficient competence in this field [21, 47]. Although nurses believed that they had the basic competencies to work during disasters, they were not adequately prepared for such events due to several reasons. These included the lack of continuity and repetition of disaster risk management training, as well as managers’ lack of commitment to disaster risk management maneuvers and training. Managers often do not take training seriously and do not provide facilities and equipment for practical training. However, Hamid et al. emphasized the critical role of managers in disaster risk management, including their commitment to providing facilities and resources, leading and coordinating educational activities, and creating motivation and dynamism among nurses [48]. The lack of skills and competencies of nurses during disasters is more related to reporting methods and access to information, incident command system, knowledge about epidemiological and biological factors, communication, and teamwork [49, 50]. The study identified several barriers to effective disaster risk management, including the unavailability of evidence, a shortage of disaster nursing professionals, and a lack of use of research evidence to prepare clinical evidence-based guidelines for disaster risk management. Despite many nurses’ involvement in disasters and their efforts to conduct research and share lessons learned, the same problems are often experienced in subsequent calamities [51]. It appears that the knowledge gained from research is not being effectively transferred to planners, policymakers, and nurses, or there is a lack of desire to use this evidence, or the working environment is not ready to accept research-based evidence [52].

Participants in the study reported experiencing more ethical challenges and concerns during disasters than in routine work environments. Gustavsson et al. also found that nurses faced moral dilemmas related to health and well-being, poor preparedness levels, limited resources, and challenges related to triage during disasters [53]. Nurses in the study stated that ethical behavior during disasters had many complications, and creating ethical challenges was inevitable. They encountered a range of challenges in their work, with some of them being familiar and routine while others were new and unfamiliar. For example, in high-risk situations, healthcare workers may be faced with challenging decisions about treatment priority, waitlists, and care allocation. Additionally, limited resources for providing care can further complicate these decisions and add to the burden of working in a stressful environment [54].

Many nurses leave their jobs due to their inability to make ethical decisions in critical situations. This distress is related to their insufficient preparation, lack of ethical guides and policies, and a lack of knowledge to manage challenging ethical situations [55]. Researchers emphasize that nurses must be well trained and prepared to manage ethically challenging situations [53]. Another challenge faced by nurses in the study was their concerns about their own health and that of their families during disasters. Many nurses faced a moral dilemma in which they had to balance their health with the health of their patients and their professional commitment. The lack of financial and psychological support for nurses aggravated this problem, leading to moral tension and stress that caused some nurses to leave their jobs [56]. It is possible that the challenges faced by nurses in this study are similar to those in other countries, but the solutions and strategies should be tailored to the context and structure of Iran.

## Limitation

The present study provides insights into the unique experiences of nurses in southeast Iran. However, it is important to note that the results may not be generalizable to all cultures and contexts. The experience of disaster risk management is influenced by cultural, economic, social, and educational factors. Therefore, it is recommended to conduct similar studies in other communities to gain a more comprehensive understanding of nurses’ experiences in disaster response.

## Conclusion

The study highlighted numerous challenges faced by nurses in disaster response, which result from environmental, managerial, individual, and educational factors. To address these challenges, it is recommended that managers establish positive relationships with nurses based on respect, understanding, and support. Plans should be developed to improve academic and continuous training, taking into account the needs and appropriate facilities and equipment, and training should be more practical. It is also important to assess the non-daily time of practice as well. Determining the challenges that nurses face during disasters is essential for improving disaster response efforts, promoting disaster preparedness, ensuring appropriate care for patients, and reducing emotional fatigue among nurses. Finally, nursing leaders, healthcare policymakers and governments should use these findings to better support the nursing workforce in disasters.

## Data Availability

The datasets used and/or analyzed during the current study are available from the corresponding author on reasonable request.

## Abbreviations

PTSD: Post-traumatic stress disorder
ED: Emergency department
